# Critical thinking skills of Chinese students: a meta-analysis of individual studies between 2002 and 2025

**DOI:** 10.3389/fpsyg.2026.1856476

**Published:** 2026-07-01

**Authors:** Weiwei Qu, Ying Chang, Huiyong Fan

**Affiliations:** 1 College of Educational Science, Bohai University, Jinzhou, China; 2 Law School, Bohai University, Jinzhou, China; 3 Center of Psychological Research and Education, Bohai University, Jinzhou, China

**Keywords:** CCTST, Chinese students, critical thinking, critical thinking skills, meta-analysis

## Abstract

Critical thinking is widely recognized as an essential competency for 21st-century higher education and the cultivation of innovative talent. However, previous studies have yielded contradictory conclusions, leaving two key questions remain unknown: What is the overall level of critical thinking skills among Chinese students, and what accounts for the inconsistencies in prior findings? To address these questions, this meta-analysis synthesized 79 primary studies published between 2002 and 2025 that assessed Chinese students’ critical thinking skills using the California Critical Thinking Skills Test or its Chinese version, with a total sample size of 8,860 participants. The results revealed that the overall score for critical thinking skills among Chinese students was 0.426, with scores on the analysis, evaluation, and inference dimensions ranging from 0.374 to 0.461, all falling below the scale midpoint. Academic discipline demonstrated a significant moderating effect, with scores ranked from highest to lowest as follows: Medicine, Liberal Arts, Education, Engineering, and Science. This variation may partly reflect differences in discipline-specific curriculum design, learning tasks, opportunities for classroom interaction, and exposure to problem-based learning. Publication type also showed a significant moderating effect, with general journals reporting the highest effect sizes and dissertations the lowest. In contrast, the moderating effects of grade level and institution type were not significant. Some issues related with the theoretical, educational implications, and future study were also discussed.

## 1 Introduction

### 1.1 Importance

Critical thinking skills are not only a major concern in international higher education, but also an important issue in China’s national talent-development strategy. In international policy discourse, critical thinking has been widely recognized as a key 21st-century competency for helping students respond to complex social, technological, and economic challenges. For example, the OECD Learning Compass 2030 identifies critical thinking as part of students’ cognitive and metacognitive skills, together with creative thinking, learning to learn, and self-regulation (OECD, 2019). In the Chinese context, President Xi Jinping emphasized that China’s high-level scientific and technological self-reliance ultimately depends on high-level innovative talent, and that talent cultivation should place greater emphasis on scientific spirit, innovation capacity, and critical thinking (Xi, 2021). Therefore, examining Chinese students’ critical thinking skills is important not only for understanding the quality of education in China, but also for contributing to the global discussion on higher-order thinking, innovation-oriented education, and talent cultivation.

Recent cross-national evidence further highlights the global relevance of this issue. Loyalka et al. (2021) compared STEM undergraduates in China, India, Russia, and the United States and found substantial cross-national differences in students’ skill levels and learning gains. Their findings suggest that the development of critical thinking is not merely a local educational issue, but a shared challenge for higher education systems worldwide. Against this global and national context, the actual level of Chinese students’ critical thinking has attracted sustained attention from the international research community for several decades (Fan and See, 2022; Huang, 2008; Jiang et al., 2024; Tian and Low, 2011; Zhang T., 2017).

Critical thinking is a complex, multidimensional psychological construct that comprises skills, dispositions, thinking styles, and ethical dimensions (Abrami et al., 2008, 2015; Baker et al., 2021; Facione, 1990a,c; Fan and See, 2022; Liu et al., 2026; Xie et al., 2025). Although critical thinking comprises multiple components, the present study focuses specifically on critical thinking skills, which represent the measurable cognitive skill dimension of critical thinking.

Critical thinking skills are defined as a set of abilities that help individuals analyze, evaluate, and reason about information, enabling them to process information rationally and make sound judgments (Facione, 1990b; Abrami et al., 2008, 2015; Huber and Kuncel, 2016; Tan, 2020; Lombardi, 2023; Xie et al., 2025). These skills typically encompass interpretation, analysis, evaluation, inference, explanation, and self-regulation (Facione, 1990b; Abrami et al., 2008).

Research on the critical thinking skills of Chinese students presents conflicting perspectives. One body of literature suggests a deficit in these skills. For instance, Turner (2006) examined nine graduate students from mainland China and found that their critical thinking performance was significantly lower than that of their peers in the UK and other European countries. Similarly, Shaheen (2016) observed that Chinese international students face challenges in cultivating such competencies, with these competencies often remaining at a relatively low level.

Conversely, other studies indicate that Chinese students possess relatively high levels of critical thinking. Yan (2018) found that students demonstrated an understanding of the importance of critical thinking when communicating in Chinese; moreover, many Chinese international students exhibited skills comparable to those of their American peers in overseas academic contexts. Hernández (2016) reported that, prior to university, Chinese students demonstrated comparatively high levels of critical thinking skills in certain aspects, although these skills tended to stagnate or regress after they entered higher education.

Despite these studies, the actual level of Chinese students’ critical thinking skills remains inconclusive. Ke and Liang (2023) argue that there is currently no conclusive evidence demonstrating that Chinese students have lower levels of critical thinking skills than their international counterparts. In a review of 15 studies, Fan and See (2022) identified nine that compared Chinese students with those from other countries. The results were mixed: three reported higher levels of critical thinking skills among Chinese students, two indicated lower scores, and four yielded inconsistent results. Crucially, Fan and See (2022) simply summarized these studies without quantitatively synthesizing the evidence, leaving the actual level of critical thinking skills among Chinese students unclear.

To address this gap, this study conducts a meta-analysis of 79 original studies. It estimates the overall level of critical thinking skills among Chinese students and examines the moderating effects of grade level, academic discipline, institution type, and publication type as potential sources of previous inconsistencies. At the end, the theoretical and practical implications are also discussed, along with suggestions for future research.

## 2 Literature review

### 2.1 Inconsistent results

Regarding the actual level of critical thinking skills among Chinese students, findings from previous research are inconsistent and can be broadly categorized into two groups. The first body of studies suggests that Chinese students possess high levels of these skills. At the high school level, Ling and Liu (2019) administered the HEIghten™ Critical Thinking Assessment (HCT) to 6,845 students and found that their average standardized scores were significantly higher than those of American college students, indicating relatively strong critical thinking skills. Among undergraduates, Ma and Feng (2022) reported that 1,042 students from China’s top universities scored higher on the HCT than their American counterparts. Similarly, Tian et al. (2020) used the Chinese version of the California Critical Thinking Skills Test (CCTST-CV) to evaluate first- to fourth-year students majoring in Measurement and Control Technology and Instrumentation at “Double First-Class” universities, finding that their critical thinking skills were at a relatively high level.

Conversely, the second body of studies suggests that the critical thinking skills of Chinese students are relatively low. Among high school students, Zhou et al. (2007) surveyed 363 students in Xi’an using the California Critical Thinking Skills Test (CCTST) and found an average score of 10.31, which fell below the normative benchmark outlined in the CCTST manual, indicating relatively low critical thinking skills. At the undergraduate level, Loyalka et al. (2021) conducted a cross-national survey including students from China, India, Russia, and the United States and reported that Chinese STEM undergraduates scored lower than their American peers in the same year of study, suggesting a relative deficiency in higher-order thinking skills. For postgraduate students, Turner (2006) examined nine postgraduate business students enrolled in a one-year program from mainland China and found that their critical thinking performance was significantly lower than that of their counterparts in the UK and other European countries, indicating potential stagnation or underdevelopment of critical thinking skills at the graduate stage.

However, this high-versus-low distinction should be understood only as a narrative device for organizing prior findings, rather than as evidence that these studies are directly comparable. The studies reviewed above differed in measurement instruments, sample characteristics, educational stages, academic disciplines, testing contexts, and performance benchmarks. Therefore, the inconsistent conclusions in prior research may be partly attributable to methodological heterogeneity and differences in sample composition. This consideration further justifies the present meta-analysis, which aims to synthesize diverse evidence systematically and examine potential moderators rather than merely juxtaposing contradictory findings.

### 2.2 The potential moderators

Several moderating variables, including grade level, academic discipline, institution type, and publication type, may help explain the inconsistent findings regarding Chinese students’ critical thinking skills.

The selection of these moderators is theoretically informed by Bronfenbrenner’s ecological systems theory. According to this theory, individual development is embedded in a multilayered ecological system, which consists of the microsystem, mesosystem, exosystem, and macrosystem (Bronfenbrenner, 1977; Bronfenbrenner and Morris, 2007). From this perspective, the development of critical thinking skills is not determined solely by individual cognitive training, but is also shaped by students’ educational environments, instructional experiences, institutional resources, and broader social contexts. Grade level may reflect differences in students’ educational stages, curriculum demands, and accumulated learning experiences. Academic discipline can be regarded as a microsystem-level learning environment, because disciplines differ in their learning tasks, classroom interaction patterns, assessment requirements, and opportunities for reasoning practice. Institution type can be regarded as an exosystem-level factor, as differences in institutional funding, educational resources, teacher development, and curriculum arrangements may indirectly shape students’ immediate learning experiences. In addition, publication type is included as a methodological moderator, because different publication channels may reflect differences in reporting practices and publication bias. Therefore, these moderators were selected to examine both educational-ecological and methodological sources of variation in Chinese students’ critical thinking skills.

#### 2.2.1 Grade level

Grade level may moderate Chinese students’ critical thinking skill scores. Some studies suggest that students in higher grades demonstrate higher levels of critical thinking skills than those in lower grades. Wang (2021) used the CCTST to assess 173 English and Business English majors from three universities, including 50 freshmen, 47 sophomores, 44 juniors, and 32 seniors. The findings revealed that students in higher years had significantly higher critical thinking skill scores than students in lower years. Zhao et al. (2015) employed the EPP (China) Critical Thinking Skills Test to assess 2,023 students from a local undergraduate university and a research-oriented university. The results indicated that students’ critical thinking skill scores increased progressively from the first to the third year of university.

Other studies report that the critical thinking skill scores of students in higher grades are lower than those of students in lower grades. Xia and Zhong (2017) used the ETS Proficiency Profile (EPP) to assess the critical thinking skills of 1,049 students from four universities. The results showed that the critical thinking skill scores of second-year students were lower than those of first-year students. Zhou and Han (2022) employed the CCTST to assess the critical thinking skills of 45 graduate students at a university in Fujian Province. The findings indicated that first-year graduate students scored significantly higher than second-year graduate students.

Furthermore, some studies suggest that there is no significant difference in critical thinking skills among students across different grade levels. Tian et al. (2020) used the CCTST-CV to assess the critical thinking skills of first- to fourth-year students majoring in Measurement and Control Technology and Instrumentation at “Double First-Class” universities, with 63, 64, 60, and 61 students in the first, second, third, and fourth years, respectively. The results showed that their critical thinking skills did not improve as grade level increased.

#### 2.2.2 Categories of academic discipline

Academic discipline may moderate Chinese students’ critical thinking skill scores. Some studies suggest that students in STEM fields score higher than those in the humanities and social sciences. Zhao et al. (2015) used the EPP (China) Critical Thinking Skills Test to assess 2,023 students from a local undergraduate university and a research-oriented university. The results showed that students in STEM disciplines had higher critical thinking skill scores than those in the humanities and social sciences. Ren et al. (2023) employed the CCTST to evaluate the critical thinking skills of 7,088 teacher education students at S University in Liaoning Province. The findings indicated that science-focused teacher education students scored higher than their humanities-focused counterparts. Zhou et al. (2007) used the CCTST to assess 363 high school students in Xi’an, China. The results revealed that students in the science track had significantly higher levels of critical thinking skills than those in the humanities track.

Other studies argue that students in STEM fields score lower than those in the humanities and social sciences. Shen et al. (2019) used the “National Undergraduate Critical Thinking Skills Assessment” tool to assess 16,000 undergraduate students across China. The findings revealed differences in critical thinking skill scores among students from different majors, with STEM students having lower average scores than their counterparts in the humanities and social sciences.

#### 2.2.3 Type of institution

According to relevant Chinese government policies, higher education institutions can be classified into national key universities and non-key universities (Ministry of Education of the People's Republic of China, Ministry of Finance, National Development and Reform Commission, 2017). Institution type may moderate Chinese students’ critical thinking skill scores. Some studies indicate that students from key universities score higher than those from non-key universities. Shen et al. (2019) used the “National Undergraduate Critical Thinking Skills Assessment” tool to evaluate 16,000 undergraduate students nationwide. The results showed that the critical thinking skill scores of students from “Project 985″ universities, “Project 211″ universities, general four-year universities, and general four-year colleges were 63.64, 57.83, 52.29, and 48.99 points, respectively. This indicates that students from key universities outperformed their counterparts from non-key universities in terms of critical thinking skills.

Other studies suggest that students from non-key universities score higher on critical thinking skills assessments than those from key universities. Xi (2018) examined 50 students who planned to study abroad, including 25 from key universities and 25 from non-key universities, and found that students from non-key universities scored significantly higher on critical thinking skills assessments than those from key universities. Zhang and Shen (2018), in an empirical study based on the “2016 National Undergraduate Skills Assessment,” pointed out that the value-added scores for critical thinking skills among students from some key universities were not always higher than those of students from non-key universities.

#### 2.2.4 Type of publication

Publication type may moderate Chinese students’ critical thinking skill scores. Methodological studies indicate that peer-reviewed journals tend to publish research with statistically significant results. This bias may lead to the overrepresentation of studies reporting significant findings, while studies reporting non-significant results may remain unpublished or overlooked (Borenstein et al., 2009; Card, 2012; Rosenthal, 1979).

A study published in a peer-reviewed journal investigated the critical thinking skills of university students and reported a mean score of 0.556 (SD = 0.09) (Xiao and Tan, 2020). An unpublished dissertation similarly examined the critical thinking skills of university students and reported a mean score of 0.366 (SD = 0.069) (Wang, 2008). The standardized mean difference between Wang (2008) and Xiao and Tan (2020) was calculated as follows: (0.366–0.556) × 2 / (0.069 + 0.09) = −2.39. According to Cohen’s (1988) criteria, this represents a large effect size, indicating that the scores reported in the peer-reviewed journal article were substantially higher than those reported in the unpublished dissertation.

### 2.3 The purpose and research questions of this study

The primary objective of this study is to estimate the overall level of critical thinking skills among Chinese students and to identify potential reasons for inconsistencies in prior research findings. Specifically, the research questions addressed in this paper are as follows:

*Q1*: What is the true level of critical thinking skills among Chinese students?

*Q2*: Is the moderating effect of grade significant?

*Q3*: Is the moderating effect of major types significant?

*Q4*: Is the moderating effect of university types significant?

*Q5*: Is the moderating effect of publication types significant?

## 3 Methods

### 3.1 CCTST

#### 3.1.1 Two versions of CCTST

The CCTST, developed in the 1990s (Facione, 1990a; Facione, 1991; Facione and Facione, 1994), consists of five subscales: analysis, evaluation, inference, deductive reasoning, and inductive reasoning. It includes 34 multiple-choice items scored dichotomously as 0 or 1. The test has demonstrated good reliability and validity (Facione, 1990b; Facione, 1991; Facione and Facione, 1994; Bielinska-Kwapisz and Brown, 2013).

According to the recommended performance assessment criteria in the CCTST Test Manual, raw scores of 0–7, 8–12, 13–18, 19–23, and 24 or higher on the 34-item CCTST indicate “Not Manifested,” “Weak,” “Moderate,” “Strong,” and “Superior” levels of critical thinking skills, respectively (Insight Assessment, 2013, p. 29). In this study, raw scores were divided by 34 and converted into standardized mean scores ranging from 0 to 1. Accordingly, the corresponding standardized ranges were 0–0.206, 0.235–0.353, 0.382–0.529, 0.559–0.676, and 0.706–1.000, respectively.

The CCTST-CV was developed by Luo and Yang through the translation and adaptation of the CCTST (Luo and Yang, 2002). The scale comprises five dimensions: analysis, evaluation, inference, inductive reasoning, and deductive reasoning. It includes a total of 34 items and is scored dichotomously as 0 or 1.

The CCTST-CV has demonstrated acceptable reliability in previous Chinese validation studies. In terms of test–retest reliability, Luo (2002) reported that the correlation between the total scores of 186 participants measured at two time points one month apart was significant, *r* = 0.63, *p* < 0.01, indicating acceptable temporal stability. In addition, split-half reliability was examined based on the structure of the five subscales. The split-half reliability coefficients were 0.75 and 0.80, respectively, both of which were statistically significant, *p* < 0.01. These findings suggest that the CCTST-CV has acceptable reliability for assessing Chinese students’ critical thinking skills (Luo, 2002; Luo and Yang, 2002).

The CCTST-CV has also shown acceptable validity, with evidence of both construct validity and criterion-related validity. Regarding construct validity, Luo and Yang (2002) conducted a three-month critical thinking instructional intervention in an educational psychology course. Students’ total CCTST-CV scores increased by 1.27 points after the intervention, *p* < 0.05, whereas no significant difference was found between two test administrations conducted one month apart in a separate retest sample. This result suggests that the scale is sensitive to changes in critical thinking skills resulting from instruction rather than merely reflecting repeated testing effects. Regarding criterion-related validity, the total CCTST-CV score was significantly correlated with students’ previous-semester GPA, *r* = 0.18, *p* < 0.05, and with Raven’s Standard Progressive Matrices scores, *r* = 0.33, *p* < 0.05. These findings support the construct and criterion-related validity of the CCTST-CV (Luo, 2002; Luo and Yang, 2002).

The meanings of the five dimensions of CCTST are as follows:

(1) Analysis

Analysis involves synthesizing and expressing the meaning of experiences, situations, data, events, judgments, dialogues, beliefs, rules, procedures, or criteria, while identifying the reasoning structures and logical relationships embedded in their expressions. It encompasses sub-skills such as categorization, decoding and clarifying meaning, as well as examining ideas, identifying arguments, and breaking them down into their constituent elements (Facione, 1990b; Luo, 2002).

(2) Evaluation

Evaluation refers to judging the credibility of statements or other forms of representation, and assessing the logical strength of the inferential relationships among these statements, descriptions, questions, or other representations. It also includes stating the outcomes of reasoning and justifying them based on the evidence, concepts, methods, criteria, or contexts on which the reasoning is grounded. Evaluation encompasses sub-skills such as assessing claims, evaluating arguments, stating results, justifying procedures, and presenting evidence (Facione, 1990b; Luo, 2002).

(3) Inference

Inference involves identifying and acquiring the necessary elements to draw reasonable conclusions, forming conjectures and hypotheses, considering relevant information, and deriving conclusions based on data, statements, principles, evidence, judgments, beliefs, opinions, concepts, descriptions, problems, or other representations. It encompasses three sub-skills: seeking evidence, generating alternative hypotheses, and drawing conclusions (Facione, 1990b; Luo, 2002).

(4) Deductive

If the truth of the premises necessarily leads to the conclusion being true, then the argument qualifies as deductive reasoning (Facione, 1990b).

Deductive reasoning refers to the process in which the assumed truth of the premises necessarily leads to the truth of the conclusion (If the assumed truth of the premises purportedly necessitates the truth of conclusion, then the argument is classified as deductive).

(5) Inductive

If the conclusion of an argument appears plausible but is not logically necessary given the truth of the premises, logicians would classify it as an inductive argument (Facione, 1990b).

Inductive reasoning refers to an argument whose conclusion is supported by the assumed truth of its premises but is not necessarily derived from them with logical certainty (If an argument’s conclusion is purportedly warranted, but not necessitated, by the assumed truth of its premises, logicians would consider the argument inductive).

### 3.2 Literature search

To improve the transparency of the literature search, the information sources were classified into three categories. First, Chinese bibliographic databases included CNKI, Wanfang, and VIP. Second, international academic databases, citation databases, and digital libraries included Web of Science, Scopus, PsycINFO, ERIC, JSTOR, and ProQuest Dissertations & Theses Global. Third, publisher full-text platforms, including Taylor & Francis Online, SAGE Journals, ScienceDirect/Elsevier, Wiley Online Library, and SpringerLink, were searched as supplementary sources to identify potentially relevant journal articles. To retrieve gray literature, additional searches were conducted in ProQuest Dissertations & Theses Global and Chinese thesis databases available through CNKI, Wanfang, and VIP.

**Figure 1:**
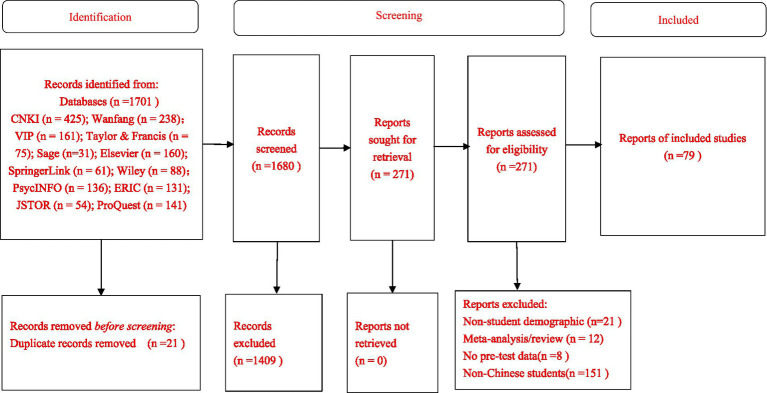
The process of literature searching. This figure (PRISMA flow diagram) was created based on the template from: Page et al. (2021). The figure has been adapted from the original template. License: CC BY. Source: https://www.prisma-statement.org.

Chinese search keywords included “critical thinking skills” (批判性思维技能 or 明辨性思维技能) and its synonyms, such as critical thinking ability (批判性思维能力), critical reasoning (批判性推理), and critical thinking (批判性思维), as well as questionnaire (or the California Critical Thinking Skills Test [加利福尼亚批判性思维技能测验]) and “Chinese students” (学生). English search keywords included “Critical Thinking Skills,” “CCTST,” “The California Critical Thinking Skills Test,” and “Students.” The search covered publications up to December 31, 2025. The search yielded 1,701 primary studies, including 824 in Chinese and 877 in English. After screening, 79 studies met the inclusion criteria. Please refer to Figure 1 for the detailed literature search process (Page et al., 2021).

### 3.3 Inclusion and exclusion criteria

(1) Participants included students from mainland China in K-12 education or higher education, excluding students from Hong Kong, Macao, and Taiwan, as well as Chinese students residing abroad.
(2) The measurement tool must be either the CCTST or the CCTST-CV.
(3) Included studies must report the following essential information: sample size, mean and standard deviation of total CCTST scores and subscale scores, as well as study characteristics, including type of institutions, publication types, academic disciplines, and grade levels.

### 3.4 Statistical computing

#### 3.4.1 Measure of effect size

This study employs the mean (M) as the effect size measure (Borenstein et al., 2009; Twenge et al., 2010; Card, 2012). In meta-analysis, when the mean is used as an effect size measure, its calculation and analytical procedures are similar to those used for other effect measures, such as r and Cohen’s d (Borenstein et al., 2009). For ease of comparison, the mean of a specific dimension is defined as the total score for that dimension divided by the number of items in that dimension. The overall mean of the scale is obtained by dividing the total scale score by the total number of items; both the CCTST and CCTST-CV contain 34 items. As a result, the value of M ranges from 0 to 1.

In the present meta-analysis, the CCTST and CCTST-CV were treated as comparable but not identical instruments. This decision was based on the fact that the CCTST-CV was translated and adapted from the CCTST, uses the same 34-item dichotomous scoring format, and measures the same core dimensions of critical thinking skills. To examine whether this decision affected the results, scale type was further included as a moderator in the moderator analysis.

#### 3.4.2 Model selection

This study adopts a random-effects model. The essential distinction between a fixed-effect model and a random-effects model lies in how they conceptualize sources of variation. The fixed-effect model assumes that all studies included in the meta-analysis share the same true effect size, with differences among observed effect sizes being attributable solely to random sampling error. In contrast, the random-effects model assumes that different studies have different true effect sizes, and that differences among observed effect sizes arise from both within-study sampling error and between-study heterogeneity (Borenstein et al., 2009; Card, 2012).

The choice between a random-effects model and a fixed-effect model primarily depends on the researcher’s objective—specifically, whether the researcher aims to generalize the findings and draw broader conclusions—rather than on the results of heterogeneity tests (Borenstein et al., 2009; Card, 2012). The fixed-effect model provides limited support for generalization beyond the included primary studies and mainly reflects the characteristics of those studies. In contrast, the random-effects model treats the set of included primary studies as a sample and seeks to generalize the results to a wider context.

#### 3.4.3 Heterogeneity test

Heterogeneity was assessed using the Q test and the *I^2^* statistic. The Q statistic is the weighted sum of squared deviations of study effect sizes from the overall effect. The *I^2^* statistic reflects the proportion of total variability across studies that is attributable to heterogeneity rather than sampling error. According to the Q test, *p* < 0.05 indicates significant heterogeneity among studies (Borenstein et al., 2009). For the *I^2^* statistic, values of 25, 50, and 75% represent low, moderate, and high levels of heterogeneity, respectively (Borenstein et al., 2009).

#### 3.4.4 Publication bias test

Publication bias refers to the phenomenon in which studies with statistically significant results are more likely to be published, whereas those with non-significant results are less likely to be published (Borenstein et al., 2009). Rosenthal’s Fail-safe N (Nfs), the funnel plot, and the trim-and-fill method are commonly used to detect publication bias (Borenstein et al., 2009; Rosenthal, 1979; Thornton and Lee, 2000). Nfs refers to the number of unpublished studies, typically with null results, that would need to be added to render the overall result statistically non-significant (Rosenthal, 1979; Thornton and Lee, 2000). The critical value for Nfs is calculated as 5 *k* + 10, where k represents the number of included studies. If Nfs exceeds the critical value, it suggests a low risk of publication bias; otherwise, it indicates a potential risk (Rosenthal, 1979; Thornton and Lee, 2000).

#### 3.4.5 Quality assessment

This meta-analysis used the Basic Quality Assessment of Primary Study (BQAPS) (Xu et al., 2024) to assess the methodological quality of the included studies. Adapted from the Cochrane Risk of Bias framework (Higgins et al., 2011), the instrument evaluates core methodological aspects, including research design, measurement reliability and validity, statistical methods, and implementation procedures.

Based on their total scores, studies were categorized into four quality levels: low quality (0–6 points), low-to-medium quality (7–12 points), medium-to-high quality (13–18 points), and high quality (19–24 points). To enhance transparency and reproducibility, detailed BQAPS assessment criteria and illustrative scoring examples are provided in the Supplementary materials (Xu et al., 2024).

#### 3.4.6 Statistical calculation tools

This study employed the Q test and the *I*^2^ statistic to assess heterogeneity. All statistical analyses were conducted using R software (R Core Team, 2023). The meta package was used for meta-regression analysis and funnel plot generation (Balduzzi et al., 2019; Schwarzer, 2025), while the metafor package was used to fit the random-effects model (Viechtbauer, 2010).

### 3.5 Sensitivity analysis

This study conducted a sensitivity analysis of the four effect size outcomes using the inverse-variance-weighted leave-one-out (LOO) method. By recalculating the weighted pooled effect sizes after sequentially excluding each study, the robustness of the meta-analytic results was examined (Viechtbauer and Cheung, 2010). The results showed that the weighted pooled effect sizes of the 62 studies were as follows: Dimension 1 (Analysis) = 0.443 (95% CI [0.439, 0.447]), Dimension 2 (Evaluation) = 0.365 (95% CI [0.362, 0.369]), Dimension 3 (Inference) = 0.327 (95% CI [0.323, 0.331]), and Overall = 0.388 (95% CI [0.385, 0.392]). The LOO analysis indicated that, after sequentially excluding each study, the maximum percentage change in the pooled effect size for each dimension ranged from 1.99 to 4.58%. The LOO estimates varied only within a narrow range: Dimension 1, [0.431, 0.457]; Dimension 2, [0.358, 0.372]; Dimension 3, [0.312, 0.340]; and Overall, [0.377, 0.399]. The point estimates for all dimensions remained in the same direction, and the 95% confidence intervals remained stable throughout the LOO process. These results suggest that the conclusions of this meta-analysis do not depend on any single study and are robust.

To examine whether the inclusion of grey literature, such as dissertations and theses, substantially affected the meta-analytic conclusions, this study compared a subsample comprising only peer-reviewed journal articles (*k* = 24) with the full sample (*k* = 62). Both samples were analyzed using a random-effects model with the DerSimonian-Laird method (DerSimonian and Laird, 1986).

Regarding the overall critical thinking score (effective *k* = 60 for the full sample), the pooled mean for journal articles only was *M* = 0.451 (95% CI [0.410, 0.492], *SE* = 0.021, *I^2^* = 95.1%), whereas the pooled mean for the full sample was *M* = 0.426 (95% CI [0.396, 0.456], *SE* = 0.003, *I^2^* = 98.4%), with a difference of only 0.025 scale units. The three sub-dimensions showed a similar trend: Analysis (*M* = 0.487 vs. 0.461, *Δ* = 0.026), Evaluation (*M* = 0.391 vs. 0.374, Δ = 0.017), and Inference (*M* = 0.475 vs. 0.449, Δ = 0.026). The 95% confidence intervals across dimensions showed substantial overlap between the two groups, with highly consistent effect directions and magnitudes. Furthermore, heterogeneity indices (*I^2^*) in both groups exceeded 92% and were comparable in level, indicating that the inclusion of dissertations did not introduce systematic bias. These results suggest that the meta-analytic conclusions of this study are robust to the inclusion of gray literature, thereby supporting the methodological decision to incorporate gray literature to mitigate publication bias (Higgins et al., 2019).

## 4 Results

### 4.1 Research characteristics

A total of 79 out of 292 primary studies met the inclusion criteria mentioned above. These 79 primary studies comprised a total of 8,860 participants. The primary studies were published between 2002 and 2025.

To improve the transparency and traceability of the included evidence, Table 1 lists the basic characteristics of all primary studies included in the meta-analysis (please refer to Supplementary Table S1 for details). Each study is identified by the first author’s surname and publication year, together with the valid sample size and publication type. Full bibliographic information for these included studies is provided in the reference list.

**Table 1:** Characteristics of the primary studies included in the meta-analysis.

Study ID	Publication year	Valid *N*	Publication type
Bai (2020)	2020	117	Dissertation
Bao (2021)	2021	246	Dissertation
Chang et al. (2023)	2023	50	Core
Chen (2017)	2017	72	Dissertation
Chen (2020)	2020	100	Dissertation
Chen (2021)	2021	98	Dissertation
Chen (2023)	2023	80	Dissertation
Dong (2018)	2018	202	Dissertation
Du (2018)	2018	210	Dissertation
Fang (2013)	2013	68	Dissertation
Feng (2014)	2014	34	Dissertation
Gao (2021)	2021	94	Dissertation
He (2021)	2021	100	Dissertation
Huang (2018)	2018	58	Dissertation
Huang (2021)	2021	86	Dissertation
Huang (2022)	2022	260	Dissertation
Jia (2022)	2022	30	Dissertation
Jiang (2012)	2012	446	Dissertation
Jiang (2018)	2018	50	Dissertation
Jin and Fang (2014)	2014	123	Core
Jou et al. (2016)	2016	60	Core
Lei (2021)	2021	100	Dissertation
Li (2020)	2020	100	Dissertation
Li et al. (2012)	2012	120	Core
Lian (2020)	2020	101	Dissertation
Liang (2021)	2021	60	Dissertation
Liao (2017)	2017	80	Dissertation
Lin (2014)	2014	46	Dissertation
Lin (2021)	2021	108	Dissertation
Ling (2018)	2018	110	Dissertation
Liu (2022)	2022	100	Dissertation
Liu (2023)	2023	108	Dissertation
Lu (2021)	2021	100	Dissertation
Luo (2002)	2002	382	Dissertation
Lv (2019)	2019	60	Dissertation
Lv (2020)	2020	50	Dissertation
Miao (2018)	2018	70	Dissertation
Nie (2020)	2020	90	Dissertation
Ouyang (2012)	2012	48	Dissertation
Pan (2019)	2021	80	Dissertation
Peng (2023)	2023	83	Dissertation
Qin (2014)	2014	50	Dissertation
Qing et al. (2010)	2010	42	Core
Su (2020)	2020	93	Dissertation
Su (2021)	2021	56	Dissertation
Sun (2020)	2020	121	Dissertation
Tang (2020)	2020	102	Dissertation
Tian (2009)	2009	119	Dissertation
Tong (2021)	2021	83	Dissertation
Wang (2008)	2008	61	Dissertation
Wang (2016)	2016	102	Dissertation
Wang (2021)	2021	192	General
Xiao and Tan (2020)	2020	25	Core
Xu (2010)	2010	101	Dissertation
Xu (2020)	2020	21	Dissertation
Xu (2023)	2023	98	Dissertation
Yang (2007)	2007	127	Dissertation
Yang (2018)	2018	65	Dissertation
Yang (2022)	2022	23	Dissertation
Yang and Chou (2008)	2008	220	Core
Yang et al. (2013)	2013	83	Core
Yao (2006)	2006	359	Dissertation
Ye (2021)	2021	91	Dissertation
Yuan (2012)	2012	460	Dissertation
Yuan et al. (2008)	2008	46	Core
Zhang R. (2017)	2017	91	Dissertation
Zhang (2018)	2018	100	Dissertation
Zhang (2022)	2022	47	Dissertation
Zhao (2012)	2012	40	Dissertation
Zhong (2019)	2019	102	Dissertation
Zhou (2022)	2022	77	Dissertation
Zhou (2023)	2023	87	Dissertation
Zhou and Han (2022)	2022	45	Core
Zhou et al. (2007)	2007	359	Core
Zhou et al. (2012)	2012	61	Core
Zhu (2021)	2021	80	Dissertation
Zhu et al. (2020)	2020	60	Core
Zhuo (2019)	2019	323	Dissertation
Zong (2018)	2018	68	Dissertation

Please see Supplementary Table S1 for details.

A total of 79 primary studies met the inclusion criteria, comprising 8,860 participants. For the moderator analyses, data were extracted at the subgroup level whenever a primary study reported results for multiple independent groups (e.g., different grade levels, academic disciplines, institution types, or publication types). Therefore, Table 2 reports both the number of primary studies contributing data to each moderator category (*k*₁) and the number of extracted subgroups used in the moderator analyses (*k*₂). Since a single primary study could contribute more than one subgroup/effect size, *k*₂ may be larger than *k*₁. N refers to the number of participants represented in each subgroup category.

**Table 2:** Study features extracted in the primary studies.

Moderators	Primary studies		Number of subgroups	
	*k_1_*	%	*k_2_*	%
Grades				
Junior middle school	4	5.06%	14	8.33%
Senior high school	44	55.70%	86	51.19%
Undergraduate	28	35.44%	63	37.50%
Postgraduate	3	3.80%	5	2.98%
Type of major				
Engineering	5	21.74%	9	16.67%
Education	5	21.74%	6	11.11%
Science	3	13.04%	9	16.67%
liberal arts	8	34.78%	25	46.30%
Medicine	2	8.7%	5	9.26%
Type of university				
Key Universities	7	43.75%	16	36.36%
Non key Universities	9	56.25%	28	63.64%
Type of publication				
General journals	1	1.27%	10	5.95%
Core Journals	13	16.46%	28	16.67%
Dissertations	65	82.28%	130	77.38%
Type of scale				
CCTST	17	22.08%	31	18.45%
CCTST-CV	60	77.92%	137	81.55%

k₁, number of primary studies contributing data to each moderator category; k₂, number of extracted subgroups used in the moderator analyses. Because a single primary study may report multiple independent subgroups, k₂may be greater than k₁. N, number of participants.

The included data covered a wide range of educational levels and institution types. Specifically, there were 14 subgroups at the junior high school level (*N* = 368), 86 at the senior high school level (*N* = 5,033), 63 at the undergraduate level (*N* = 3,290), and 5 at the postgraduate level (*N* = 169). In addition, the sample included 16 subgroups from key universities (*N* = 703) and 28 subgroups from non-key universities (*N* = 1,460). The distribution of subgroups is presented in Table 2.

### 4.2 Results of heterogeneity tests

The *Q*-test value for the total score of the CCTST was 3592.36, *p* < 0.001. For the various dimensions of the CCTST, the *Q*-test values ranged from 3401.16 to 10485.98, *p* < 0.001. The *I^2^* value for the total score of the CCTST was 98.4%, while the *I^2^* values for its sub-dimensions ranged from 97.4% to 99.4%.

According to Table 3, there is significant heterogeneity in the effect sizes of both the total score and the sub-dimensions of the CCTST. Therefore, it is necessary to conduct moderation analyses to identify the potential sources of these variations among primary studies.

**Table 3:** The estimated effect sizes and heterogeneity test results for Chinese students’ CCTST scores.

CCTST dimensions	*k*	*Mean*	*SE*	95%CI	*I* ^2^	*Q*
CT Total	60	0.426	0.003	0.396, 0.456	0.984	3592.36***
Analysis	62	0.461	0.003	0.433, 0.490	0.982	3401.16***
Evaluation	62	0.374	0.003	0.348, 0.399	0.974	2337.75***
Inference	62	0.449	0.005	0.401, 0.497	0.994	10485.98***

* *p* < 0.05, *** *p* < 0.001.

Given these extremely high *I^2^* values, the pooled mean should be interpreted with caution. Specifically, it should not be regarded as a precise or uniform estimate applicable to all Chinese students, but rather as an overall central tendency across heterogeneous samples, educational stages, academic disciplines, institutions, and publication contexts. Therefore, the subsequent moderator analyses and subgroup estimates were used to provide more context-specific interpretations.

### 4.3 Results of publication bias tests

**Figure 2:**
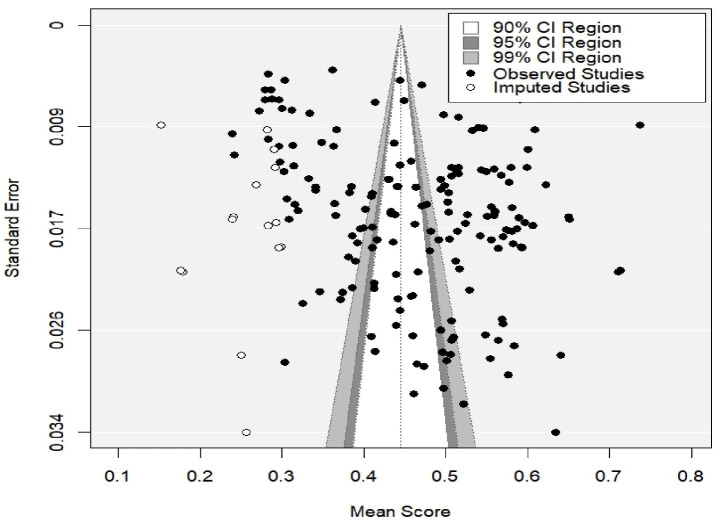
The trimmed funnel plot of the current meta analysis. The funnel plot displays the distribution of effect sizes (Mean Score) against their standard errors. The solid lines represent the 95% confidence interval region. The filled circles (●) represent the observed studies, and the open circles (○) represent the imputed studies from the trim-and-fill analysis.

This study employed multiple methods to comprehensively assess publication bias. The funnel plot (see Figure 2) revealed a certain degree of asymmetry in the distribution of effect sizes. It should be noted, however, that funnel plot asymmetry does not necessarily indicate publication bias. As Borenstein et al. (2009) and Sterne et al. (2011) noted, asymmetry can arise from multiple sources, including true between-study heterogeneity, small-study effects, and differences in methodological quality between smaller and larger studies. Given the extremely high heterogeneity observed in the present meta-analysis (*I^2^* = 98.4%), the observed asymmetry may partly reflect genuine variation in effect sizes across studies rather than selective reporting alone. Therefore, conclusions about publication bias should not be based on funnel plot inspection alone, but should be informed by convergent evidence from multiple correction methods.

The trim-and-fill analysis showed that the mean effect size before trimming was 0.462 (95% CI [0.446, 0.478]), compared to 0.371 (95% CI [0.350, 0.390]) after trimming, representing a reduction of approximately 19.6%. This non-trivial downward correction suggests that some small-study effects or publication-related bias may be present in the literature.

The fail-safe *N* (Nfs) was 133,257, far exceeding the critical threshold of 405 (5 *k* + 10 = 5 × 79 + 10 = 405), indicating that a large number of unpublished null-effect studies would be required to render the overall pooled estimate statistically non-significant (Borenstein et al., 2009; Card, 2012). It should be noted, however, that the fail-safe N and the trim-and-fill analysis address different aspects of publication bias. The fail-safe *N* mainly evaluates the stability of statistical significance, whereas the trim-and-fill analysis examines the possible influence of funnel plot asymmetry on the magnitude of the pooled estimate. Therefore, these findings should be interpreted together rather than as contradictory evidence. Although the fail-safe N suggests that the pooled result is statistically robust, the trim-and-fill correction indicates that the magnitude of the pooled estimate may still be influenced by small-study effects or publication-related bias.

To further examine the impact of publication bias, this study conducted supplementary PET-PEESE analyses (Carter et al., 2019). The PET regression analysis revealed a significantly positive intercept (*b* = 0.384, *p* < 0.001), indicating that even when extrapolating precision to infinity, the effect size remains significantly greater than zero, which supports the existence of a true effect. The PEESE-corrected effect size was 0.426 (95% CI [0.401, 0.452]).

Furthermore, this study conducted a dummy-coded random-effects meta-regression analysis using publication type as a moderator. The results showed no significant differences in effect sizes among dissertations, core journals, and general journals, *Q_M_*(2) = 2.31, *p* = 0.315, indicating that publication type did not systematically explain between-study variation in effect sizes.

Taken together, the convergent evidence from multiple methods indicates that some small-study effects or publication-related bias may be present in this literature. The bias-corrected estimates ranged from *M* = 0.371 (trim-and-fill) to *M* = 0.426 (PEESE). Researchers should interpret the uncorrected pooled estimate (*M* = 0.426) with caution and treat the bias-corrected estimates as more conservative benchmarks. Nevertheless, across all correction methods, the estimated effect sizes consistently remained below the theoretical scale midpoint of 0.5, supporting the conclusion that Chinese students demonstrate a low-to-moderate level of critical thinking skills. The core directional conclusion of this meta-analysis is therefore robust to the influence of publication bias.

### 4.4 Results of quality evaluation

Among the 79 primary studies included, no studies were rated as moderate-to-low quality (quality assessment scores: 7–12). Specifically, 18 studies (22.78%) were classified as moderate-to-high quality (scores 13–17), while 61 studies (77.22%) were rated as high quality (scores 18–24).

To examine whether study quality influenced the meta-analytic results, we conducted a meta-regression analysis using the BQAPS quality score as a continuous moderator. The results showed that the meta-regression was not significant, *F*(1, 166) = 0.071, *p* = 0.790, indicating that the quality score did not significantly predict the overall mean score of critical thinking skills among Chinese students. Therefore, variations in study quality did not systematically affect the main findings of this study.

### 4.5 Main effect

According to Table 3, the pooled mean score for Chinese students’ overall critical thinking skills was 0.426 (*k* = 60, 95% CI [0.396, 0.456]). Based on the standardized CCTST performance criteria, this score falls within the moderate range of 0.382–0.529. Nevertheless, as it remains below the scale midpoint of 0.50, Chinese students’ overall critical thinking skills can be characterized as being at a moderate-to-low level.

The average effect size (*ES*) for the analytical dimension among Chinese students is 0.461 (*k* = 62, 95% CI [0.433, 0.490]). The average ES for the evaluation dimension is 0.374 (*k* = 62, 95% CI [0.348, 0.399]). The average ES for the inference dimension is 0.449 (*k* = 62, 95% CI [0.401, 0.497]).

To facilitate the interpretation of the standardized mean scores, we added a comparison between Chinese and American undergraduates. As presented in Table 4, Chinese undergraduates scored slightly lower than American. undergraduates on the total CCTST score and the analysis dimension, with a more pronounced disadvantage in inference. In contrast, they scored slightly higher on evaluation. These findings suggest that Chinese undergraduates’ critical thinking skills are not uniformly weaker across all dimensions; rather, their relative disadvantages are mainly reflected in inference and analysis. Nevertheless, this cross-national comparison should be interpreted cautiously due to possible differences in sample composition, language, educational systems, and testing contexts.

**Table 4:** Comparison of critical thinking skills between Chinese and American undergraduates.

CCTST dimensions	Chinese undergraduates			American undergraduates			*d*
	*k*	*Mean*	*SD*	*k*	*Mean*	*SD*	
CT total	25	0.43	0.11	104	0.47	0.12	−0.34
Analysis	25	0.45	0.17	104	0.51	0.16	−0.37
Evaluation	25	0.39	0.15	104	0.35	0.14	0.28
Inference	25	0.44	0.16	104	0.60	0.19	−0.85

The d values represent the comparison of Chinese undergraduates versus American undergraduates. The data of American undergraduates come from our another work. This table serves only as a rough comparison for easy understanding. Please refrain from overgeneralizing or over-interpreting the results.

### 4.6 Results of the moderator analyses

#### 4.6.1 Moderating effect of grade level

Based on grade level, the primary studies were categorized into four groups: junior high school students (*k* = 14), senior high school students (*k* = 86), undergraduate students (*k* = 63), and postgraduate students (*k* = 5). The between-group difference test yielded *Q*_between_ = 4.45, *p* > 0.05, indicating that the moderating effect of grade level is not significant.

#### 4.6.2 Moderating effect of academic discipline

Based on academic discipline, the primary studies were categorized into five groups: engineering (*k* = 9), education (*k* = 6), science (*k* = 9), liberal arts (*k* = 25), and medicine (*k* = 5). The between-group difference test yielded *Q*_between_ = 12.46, *p* < 0.05, indicating a significant moderating effect of academic discipline. The mean scores across disciplines, ranked from highest to lowest, were as follows: medicine (*M* = 0.548, *SE* = 0.047), liberal arts (*M* = 0.509, *SE* = 0.012), education (*M* = 0.492, *SE* = 0.009), engineering (*M* = 0.468, *SE* = 0.025), and science (*M* = 0.448, *SE* = 0.021).

To further quantify the extent to which the examined moderators explained between-study heterogeneity, dummy-coded random-effects meta-regression analyses were conducted. The results showed that academic discipline significantly explained part of the between-study heterogeneity, *Q_M_*(4) = 17.20, *p* = 0.002, pseudo *R*^2^ = 30.17%. In contrast, grade level, *Q_M_*(3) = 2.98, *p* = 0.394, institution type, *Q_M_*(1) = 1.44, *p* = 0.229, publication type, *Q_M_*(2) = 2.31, *p* = 0.315, and scale type, *Q_M_*(1) = 0.36, *p* = 0.549, did not show significant explanatory effects. These findings suggest that, among the examined moderators, academic discipline was the most important identifiable source of heterogeneity, although substantial residual heterogeneity remained.

#### 4.6.3 Moderating effect of institution type

Based on institution type, the primary studies were categorized into two groups: non-key universities (*k* = 28) and key universities (*k* = 16). The between-group difference test yielded *Q*_between_ = 1.8, *p* > 0.05, indicating that the moderating effect of institution type is not significant.

#### 4.6.4 Moderating effect of publication type

Based on publication type, the primary studies were categorized into three groups: general journals (*k* = 10), core journals (*k* = 28), and dissertations (*k* = 130). The between-group difference test yielded *Q*_between_ = 15.01, *p* < 0.001, indicating a significant moderating effect of publication type. The mean scores across publication types, ranked from highest to lowest, are as follows: general journals (*M* = 0.504, *SE* = 0.009), core journals (*M* = 0.479, *SE* = 0.017), and dissertations/theses (*M* = 0.456, *SE* = 0.010).

#### 4.6.5 Moderating effect of scale type

Based on scale type, the primary studies were categorized into two groups: CCTST and CCTST-CV. The results showed that the mean score for studies using the CCTST was 0.451, whereas the mean score for studies using the CCTST-CV was 0.465. The between-group difference test was not significant, Q_between_ = 0.36, *p* > 0.05, indicating that scale type did not significantly moderate Chinese students’ critical thinking skills scores.

## 5 Discussion

This study extends previous research in two ways. First, we provide an overall estimation of the true level of critical thinking skills among Chinese students. Second, using the meta-analysis method, we simultaneously examine the potential moderating effects of grade level, institution type, academic discipline, and publication type on the critical thinking skills of Chinese college students.

### 5.1 Overall estimation of critical thinking skills

The primary and most significant finding of the current study is the meta-analyzed data for the critical thinking skills score of Chinese students (see Table 3). The averaged total score of critical thinking skills of Chinese students is 0.426 (*k* = 60, 95% CI [0.396, 0.456]). The scores of the three subscales range from 0.374 to 0.461.

The single pooled estimates help us capture the central tendency of Chinese students’ critical thinking skills. The average scores for both the total CCTST (California Critical Thinking Skills Test) and its subscales were below 0.5 (half of the total possible score), suggesting that the overall CCTST performance of the Chinese student population may be at a low-to-moderate level. Furthermore, those averaged scores can serve as a reference benchmark to explain new CCTST scores in the future.

However, it should be noted that these single pooled estimates are computed based on a dataset with extreme heterogeneity. Therefore, subgroup-based estimates (Table 5) are better suited for explaining specific scores of the CCTST.

**Table 5:** Results of moderator analyses (based on total score).

Moderators	*k*	*Mean*	*SE*	95%CI	*Q*_b_
Grades					
Junior middle school	14	0.442	0.037	0.369, 0.515	4.45
Senior high school	86	0.451	0.012	0.427, 0.475	
Undergraduate	63	0.481	0.012	0.456, 0.505	
Postgraduate	5	0.486	0.029	0.430, 0.543	
Type of major					
Science	9	0.448	0.021	0.407, 0.489	12.46*
Engineering	9	0.468	0.025	0.419, 0.518	
Medicine	5	0.548	0.047	0.455, 0.640	
Literature	25	0.509	0.012	0.486, 0.532	
Education	6	0.492	0.009	0.475, 0.509	
Type of university					
Key Universities	16	0.507	0.017	0.473, 0.541	1.80
Non key Universities	28	0.471	0.022	0.427, 0.515	
Type of publication					
General journal	10	0.504	0.009	0.487, 0.521	15.01***
Core Journals	28	0.479	0.017	0.446, 0.512	
Dissertation	130	0.456	0.010	0.436, 0.475	
Type of scale					
CCTST	31	0.451	0.016	0.420, 0.482	0.36
CCTST-CV	137	0.465	0.011	0.444, 0.486	

* *p* < 0.05, *** *p* < 0.001.

In addition, the non-significant moderating effect of scale type suggests that the pooled estimates were not substantially influenced by whether the original CCTST or the CCTST-CV was used. However, because substantial heterogeneity remained, the overall pooled mean should be interpreted as a summary of the central tendency rather than as a uniform estimate applicable to all student groups, disciplines, or educational contexts.

### 5.2 Significant moderating effect of academic discipline

This study found a significant moderating effect of academic discipline on critical thinking skills, with scores ranked from highest to lowest as follows: medicine, liberal arts, education, engineering, and science. This finding extends the existing literature but is inconsistent with some previous studies (Zhao et al., 2015; Ren, 2023; Zhou et al., 2007; Dong and Liu, 2013), which reported that science students had higher critical thinking skill scores than liberal arts students. However, our finding is supported by Loyalka et al. (2021). There are two possible reasons why science and engineering students have lower critical thinking skills scores than liberal arts students.

First, students in science and engineering disciplines may receive relatively fewer opportunities for critical thinking skills training. Su (2019) pointed out that critical thinking skills training is notably deficient in engineering education. Most instructors of engineering courses generally lack both the awareness and the skills needed to teach critical thinking, making it difficult to effectively integrate critical thinking skills training into their curricula. Ren, Liu, and Wang (2023) further noted that current research on instructional interventions related to critical thinking skills is primarily concentrated in fields such as medicine, foreign languages, and educational technology. There remains a relative lack of both research and practice in STEM fields, which are closely linked to national scientific and technological innovation.

Second, disciplines including science and engineering, the humanities, medicine, and education may not explicitly designate the cultivation of critical thinking skills as a mandatory objective. National policy documents have not included critical thinking skills in the teaching standards of various disciplines (Ministry of Education of the People's Republic of China, 2018), which may have resulted in a lack of clear guidance and requirements for cultivating critical thinking skills across different subjects. Although educational investment in science and engineering disciplines is relatively high, the development of critical thinking skills has not received sufficient emphasis (Dong and Liu, 2013). Teaching methods in these disciplines often prioritize knowledge transmission and focus on memorizing basic concepts and formulas, with insufficient emphasis on students’ in-depth understanding of the principles underlying knowledge and skills. Consequently, students may struggle to flexibly apply what they have learned to real-world problems (Dong and Liu, 2013). In contrast, liberal arts students are exposed to a broader range of knowledge upon entering university. Their learning content encompasses ideological theories and educational concepts, and extensive reading, along with interdisciplinary exposure, contributes to the cultivation of critical thinking skills (Ren, 2023).

From the perspective of Bronfenbrenner’ s ecological systems theory, academic discipline can be understood as an important microsystem in which students directly participate (Bronfenbrenner, 1977; Bronfenbrenner and Morris, 2007). Different disciplines provide students with distinct learning tasks, classroom interaction patterns, assessment requirements, and opportunities for reasoning practice. Therefore, the disciplinary differences found in this study should not be interpreted merely as differences in students’ individual abilities, but rather as differences in the immediate learning ecologies created by different disciplines.

### 5.3 Significant moderating effect of publication type

This meta-analysis found that publication type had a significant moderating effect, with journal articles reporting larger effect sizes than dissertations. This finding is consistent with methodological evidence suggesting that peer-reviewed journals are more likely to publish studies with stronger or statistically significant results (Borenstein et al., 2009; Card, 2012; Rosenthal, 1979; Thornton and Lee, 2000).

However, the significant moderating effect of publication type should be interpreted cautiously. Although journal articles reported higher effect sizes than dissertations, this pattern is consistent with well-documented methodological evidence that peer-reviewed journals are more likely to publish studies with statistically significant or stronger results (Borenstein et al., 2009; Card, 2012; Rosenthal, 1979; Thornton and Lee, 2000). It should be noted, however, that funnel plot asymmetry does not constitute direct evidence of publication bias. As Borenstein et al. (2009) and Sterne et al. (2011) noted, asymmetry can arise from multiple sources beyond publication bias, including true between-study heterogeneity, small-study effects, and differences in methodological quality between smaller and larger studies. Given the extremely high heterogeneity observed in the present meta-analysis (*I^2^* = 98.4%), the observed funnel plot asymmetry may partly reflect genuine variation in effect sizes across studies rather than selective reporting alone. The comprehensive publication bias analyses, including the Fail-safe N, trim-and-fill, and PET-PEESE, suggest that while some small-study effects or publication-related bias may be present, the core directional conclusion that Chinese students’ critical thinking skills are below the theoretical scale midpoint remains robust across all correction methods.

### 5.4 Insignificant moderating effect of grade level

This study found that the moderating effect of grade level on critical thinking skills was not significant. This result is consistent with Tian et al. (2020), who found no significant correlation between critical thinking skill scores and grade level, suggesting that students’ critical thinking skills do not improve significantly as they advance to higher grades. However, this finding is inconsistent with the results reported by Sun and Qi (2020).

This result may be due to two potential explanations. First, students across all grade levels have limited opportunities to receive training in critical thinking skills. Previous studies have pointed out that Chinese students lack critical thinking skills and have insufficient opportunities for training (Tian and Low, 2011; Lu and McKechnie, 2017). For K-12 students in China, their learning is primarily exam-oriented (Durkin, 2008; Lu and McKechnie, 2017). They spend a great deal of time on repetitive exercises (Zheng, 2013; Zhai and Fan, 2021; Ma and Yang, 2022). As for university students, they similarly face a shortage of opportunities for critical thinking skills training. For instance, Sun et al. (2013), in their investigation of some universities in the Nanjing area, found that critical thinking skills courses had low coverage. Some institutions even lacked relevant curricula entirely, and where such courses were offered, they mostly existed as general electives.

Second, training in critical thinking skills is not a mandatory objective across all grade levels (Ministry of Education of the People's Republic of China, 2011). Before 2020, national curriculum standards did not designate critical thinking skills as a compulsory teaching goal. As a result, teachers were not required to cultivate students’ critical thinking skills in their instructional processes. The situation at the higher education level is similar. It was not until 2018 that the Ministry of Education of China emphasized the importance of advanced curricula; however, critical thinking skills were still not designated as a mandatory objective in higher education (Ministry of Education of the People's Republic of China, 2018).

### 5.5 Insignificant moderating effect of institution type

This study also found that the moderating effect of institution type was not significant. This result is inconsistent with previous findings (e.g., Shen et al., 2019). Two plausible explanations for this discrepancy are as follows:

First, neither type of institution has established the cultivation of critical thinking skills as a mandatory educational objective. It was not until 2018 that the Chinese Ministry of Education emphasized that curricula should focus on higher-order competencies; however, the cultivation of critical thinking skills has still not been designated as a compulsory goal for higher education (Ministry of Education of the People's Republic of China, 2018). Consequently, teachers have not prioritized fostering critical thinking skills in their teaching practice. Although key universities have advantages in terms of faculty qualifications, they remain deficient in the teaching of critical thinking skills. More critically, many higher education institutions, especially those with engineering programs, lack systematic teacher training in critical thinking skills instruction, making it difficult to effectively integrate critical thinking skills training into the curriculum (Su, 2019). This situation indicates that the development of critical thinking skills is not only a weakness in key universities but also a common shortcoming across other institutions. Regardless of institution type, the scarcity of teaching resources and qualified instructors for critical thinking skills may directly hinder the improvement of students’ critical thinking skills.

Second, opportunities for training in critical thinking skills are generally insufficient across both key and non-key universities. At both key and non-key universities, courses focused on critical thinking skills development are limited in scope, and the percentage of students taking these courses is low. Some schools even lack such courses (Qian, 2018; Sun et al., 2013). While key universities hold certain advantages in terms of resources, they do not demonstrate a significant difference from non-key universities in terms of actual instructional practices for critical thinking skills and the effectiveness of skill development.

### 5.6 Theoretical and educational implications

#### 5.6.1 The theoretical implication of this study

By examining one of the world’s largest standardized education systems (China), this study provides critical evidence for the global discourse on the tension between high-stakes testing and the cultivation of critical thinking skills.

This study extends previous review studies. Previous studies did not quantitatively synthesize evidence on the critical thinking skills of Chinese students (Fan and See, 2022; Ke and Liang, 2023). To address this issue, this study employed a meta-analytic approach, analyzing 79 primary studies using the California Critical Thinking Skills Test. It estimated the population-level scores of Chinese students, which can serve as reference data for future studies.

This study further extends prior research on the influence of academic discipline on critical thinking skills. Previous studies have found significant differences in the value-added effects on critical thinking skills across disciplines, with the order from highest to lowest being liberal arts, medicine, science, and engineering (Zhang and Shen, 2018). The present study includes a more comprehensive range of disciplines, including science, engineering, medicine, liberal arts, and education, and uses a larger sample size. It reveals that critical thinking skill scores, ranked from highest to lowest, are as follows: medicine, liberal arts, education, engineering, and science, thereby providing more robust evidence.

This study challenges the prediction of human capital theory, which posits that investment in education leads to the development of human capital attributes (Zhang et al., 2022; Blair, 2011; Brown, 2006; Leoni, 2025; Sycheva et al., 2019). According to this theory, disciplines receiving higher levels of financial support should have higher critical thinking skill scores. However, our findings contradict this prediction, as disciplines receiving lower levels of financial support actually achieved higher scores. This suggests that financial support may not directly influence students’ critical thinking skills; rather, the explicit setting of critical thinking skills objectives and the availability of training opportunities may mediate the relationship between funding and critical thinking skills.

#### 5.6.2 The practical implications for education

The findings of this study hold significant implications for educational practice globally.

First, courses related to critical thinking skills should be offered in middle schools and universities, or critical thinking skills content should be integrated into existing courses. Xie et al. (2025) analyzed key educational policy documents in China, including the framework for core competencies of Chinese students and the 2020 Compulsory Education Curriculum Standards. They found that although these documents explicitly emphasize the importance of critical thinking skills, related courses are still lacking at both the middle school and university levels. In Chinese middle schools, there are few dedicated courses on critical thinking skills; only sporadic knowledge points appear in certain subjects. For instance, our review of all 11 mathematics textbooks published by the People’s Education Press revealed that critical thinking skills content is minimal. Only the eighth-grade volume (2013 edition) briefly raises the question “Why prove?” without introducing foundational concepts of proof, and the high school compulsory volume 1 (2016 edition) provides definitions of sufficient and necessary conditions, as well as universal and existential quantifiers. At the university level, courses related to critical thinking skills are also not widely offered. Qian’s (2018) launch of the Logic and Writing course at Tsinghua University’s School of Economics and Management was praised as a rare highlight. Isolated knowledge points can hardly meet students’ needs for systematic learning of critical thinking skills.

Second, teachers should proactively incorporate knowledge related to critical thinking skills into their instruction. Teachers’ awareness of and competence in critical thinking skills directly influence the development of students’ critical thinking skills (Li and Wang, 2015). Therefore, teachers themselves must first receive training in critical thinking skills and then integrate critical thinking into subject-specific teaching (Miao, 2007). Teachers should cultivate students’ abilities in analysis, evaluation, and inference through practices such as hands-on activities and interactive exchanges (Dong and Liu, 2013). Taking mathematics instruction as an example, teachers should first create instructional contexts and organize students to engage in collaborative inquiry, deepening their understanding of knowledge through discussion and debate. Teachers should guide students to analyze and evaluate problems from multiple perspectives in a dialectical manner, thereby fostering their spirit of independent thinking and questioning. Teachers should encourage students to apply the thinking methods they have learned to reason through new problems and justify their solutions, thereby helping students apply critical thinking skills to solve real-world issues (Li, 2018; Wang, 2023).

Third, students should proactively learn critical thinking skills, rather than simply focusing on exam preparation. Mao Zedong serves as an excellent example of self-directed cultivation of critical thinking skills. In 1912, while studying independently at the Hunan Provincial Library, Mao read books on logic, such as *A System of Logic, Ratiocinative and Inductive* (Mill, 1875) and *A Brief Exposition of Logic* [名学浅说 in Chinese] (Chen, 2018). In the spring of 1938, Mao read *Logic and Logic Studies* [逻辑与逻辑学 in Chinese] (Chen, 2013). In 1959, Mao Zedong reviewed the *Collected Papers on Logic* compiled by Jiang Chunfang and others, which contained 150 papers. Later, he read 11 pre-1949 monographs on logic (Chen, 2018). Mao Zedong’s deep engagement with works on logic significantly enhanced his critical thinking skills. This is evidenced by the series of new concepts and ideas he introduced in his own writings and articles.

Fourth, critical thinking skills should be incorporated into the assessment frameworks of key examinations such as the senior high school entrance exam (Zhongkao), the national college entrance exam (Gaokao), and postgraduate admission exams. These high-stakes examinations play a guiding role in educational practice. Previously, the lack of emphasis on critical thinking skills was largely due to its absence from these assessments. Including it in examinations would serve as a strong positive signal and effectively steer educational focus. The *Overall Plan for Deepening Educational Evaluation Reform in the New Era* explicitly calls for steadily advancing the reform of entrance examinations, establishing an assessment system that fosters students’ all-round moral, intellectual, physical, aesthetic, and labor development, and reducing reliance on rote memorization and “mechanical drilling” (Central Committee of the Communist Party of China and State Council, 2020). Integrating critical thinking skills assessment into major exams like the Zhongkao and Gaokao aligns fully with these policy objectives.

Fifth, higher education institutions should adopt evidence-based instructional approaches to enhance students’ critical thinking skills. Given the significant moderating effect of academic discipline, critical thinking skills education should not rely only on general courses or broad policy statements. Instead, it should be embedded into discipline-specific teaching. In particular, science and engineering programs may benefit from instructional approaches such as Problem-Based Learning and Project-Based Learning, which can provide students with more opportunities to analyze complex problems, evaluate evidence, make inferences, justify conclusions, and apply knowledge to real-world contexts (Hmelo-Silver, 2004; Thomas, 2000; Abrami et al., 2015). At the policy level, critical thinking skills should be incorporated into curriculum design, teacher professional development, and assessment rubrics as an explicit and assessable learning outcome.

## 6 Limitations and issues requiring future research

This study has the following limitations: First, this study only included Chinese students’ scores obtained using the CCTST or CCTST-CV. Although the CCTST-CV is a translated and adapted version of the CCTST and the scale-type moderator analysis showed no significant difference between the two instruments, the use of this single instrument family may still limit cross-validation with other critical thinking measures. Other instruments such as the *Watson-Glaser Critical Thinking Appraisal* (WGCTA) (Liu, 2015) and the *Cornell Critical Thinking Test* (CCTT) (Leng and Du, 2022) have also been used to some extent with Chinese students, but their application remains limited, with only about ten relevant studies identified.

Second, in the moderation analysis, the number of primary studies in certain categories was relatively small. For instance, when examining the moderating effect of academic discipline on the analytical, evaluative, and inferential dimensions, the sample sizes were insufficient for the following groups: science (*k* = 2), engineering (*k* = 3), medicine (*k* = 3), liberal arts (*k* = 4), and education (*k* = 1). A limited number of primary studies may reduce the accuracy of the findings and potentially lead to erroneous conclusions.

Third, the comparison of grade-level differences is not based on longitudinal data but rather on cohort comparisons across different batches of participants. The lack of significant grade-level differences may be attributed to unaccounted confounding factors.

The main issues that need to be studied in the future are as follows:

First, future research should improve the measurement and cross-validation of Chinese students’ critical thinking skills. Because the present meta-analysis only included studies that used the CCTST or CCTST-CV, future studies should compare results from multiple instruments, such as the WGCTA, CCTT, and CCTST, and examine whether existing scales fully capture important dimensions of critical thinking skills, including self-regulation and culturally relevant dimensions.

Second, longitudinal studies are needed to examine grade-level differences more accurately. Although the moderating effect of grade level was not significant in this study, the result was mainly based on comparisons across different groups of students. Future research should track the same cohort over time to determine whether critical thinking skills improve, stagnate, or decline with grade progression.

Third, more discipline-specific studies are needed. Although academic discipline showed a significant moderating effect, the number of studies in some fields, especially science, engineering, medicine, and education, was relatively limited. Future research should further examine how discipline-specific curriculum design, instructional methods, assessment requirements, and learning opportunities influence students’ critical thinking skills.

Fourth, future research should further investigate the effect of institution type. The non-significant moderating effect of institution type in this study is inconsistent with some previous findings, such as Zhang and Shen (2018). Future studies should examine whether this inconsistency is related to measurement tools, sample composition, institutional classification, curriculum implementation, teacher support, or actual learning opportunities.

Fifth, future research should pay more attention to publication characteristics and evidence quality. Since publication type showed a significant moderating effect, future studies should include both journal articles and grey literature to reduce publication bias. Meanwhile, primary studies should report complete sample information, means, standard deviations, measurement tools, and study characteristics to improve the quality of future evidence synthesis.

## 7 Conclusion

Through a meta-analysis of a total sample of 8,860 Chinese students, this study systematically assessed the level of critical thinking skills among Chinese students and identified moderating factors contributing to inconsistencies in previous research.

The main findings indicate that Chinese students’ overall critical thinking skills were at a low-to-moderate level. Academic discipline and publication type showed significant moderating effects, whereas grade level and institution type did not. In particular, the significant moderating effect of academic discipline suggests that the development of critical thinking skills is closely related to disciplinary learning environments, curriculum structures, and opportunities for reasoning practice.

This study contributes to the field of education by providing a quantitative synthesis of Chinese students’ critical thinking skills and by identifying potential factors that may explain inconsistencies in previous findings. It also provides empirical evidence for higher education curriculum development, instructional strategy design, and critical thinking skills assessment.

The findings of this study offer several implications for higher education practice. First, in terms of curriculum development, universities should provide students with more opportunities to engage in active learning through problem-based learning, case discussions, and interdisciplinary projects. Second, in terms of instructional strategies, instructors should guide students by providing structured support, timely feedback, and opportunities for reflection to foster students’ critical thinking skills. Third, in terms of assessment design, educational assessments should incorporate higher-order reasoning and analytical skills, rather than relying solely on memory or repetitive tasks. These findings should be interpreted cautiously, particularly in cross-national contexts, because cultural backgrounds, language contexts, educational systems, and measurement differences may influence students’ performance in critical thinking skills.

Beyond the Chinese context, the findings of this study also have implications for global higher education. As many countries seek to strengthen students’ higher-order thinking skills, the Chinese case provides useful evidence on how disciplinary environments, curriculum structures, and examination-oriented educational cultures may shape the development of critical thinking skills. Overall, the development of critical thinking skills remains essential for 21st-century learning, innovation-oriented talent cultivation, and students’ ability to respond to complex social, technological, and global challenges.
