# Supplementary material for: Critical thinking skills of Chinese students: a meta-analysis of individual studies between 2002 and 2025
Source: Front Psychol. 2026 Jul 1;17:1856476. doi: 10.3389/fpsyg.2026.1856476 (PMC13368918; doi:10.3389/fpsyg.2026.1856476)
Supplement: Supplementary file 2 [file Table_2.docx]

**This file lists all 79 primary studies included in the current meta analysis**

Bai, Y. (2020). *Teaching practice for purposefully cultivating critical thinking ability in senior high school biology* (Master’s thesis, Shanxi Normal University). https://doi.org/10.27287/d.cnki.gsxsu.2020.000996

[白玉萍. (2020). *高中生物有目的地培养批判性思维能力的教学实践*  (硕士学位论文, 山西师范大学). 硕士 https://link.cnki.net/doi/10.27287/d.cnki.gsxsu.2020.000996]

Bao, J. (2021). A study on classroom questioning in high school English reading lessons based on critical thinking (Master's thesis, Yili Normal University). [https://doi.org/10.27808/d.cnki.gylsf.2021.000017](https://doi.org/10.27808/d.cnki.gylsf.2021.000017.)

[包金梅. (2021). *基于批判性思维的高中英语阅读课课堂提问研究* (硕士学位论文, 伊犁师范大学). 硕士 [https://link.cnki.net/doi/10.27808/d.cnki.gylsf.2021.000017]](https://link.cnki.net/doi/10.27808/d.cnki.gylsf.2021.000017.])

Chang, J. L., Hung, H. T., & Yang, Y. T. C. (2024). Effects of an annotation‐supported Socratic questioning approach on students' argumentative writing performance and critical thinking skills in flipped language classrooms. *Journal of Computer Assisted Learning,*40(1), 37-48.

Chen, C. (2021). *Cultivating critical thinking in high school English reading teaching* (Master's thesis, Southwest University). [https://doi.org/10.27684/d.cnki.gxndx.2021.002268](https://doi.org/10.27684/d.cnki.gxndx.2021.002268.)

[陈辰. (2021). *高中英语阅读教学中的批判性思维培养* (硕士学位论文, 西南大学). 硕士 https://link.cnki.net/doi/10.27684/d.cnki.gxndx.2021.002268 ]

Chen, X. (2023). *Effects of a biology Predict–Observe–Explain (POE) instructional strategy on high school students’ critical thinking* (Master’s thesis, Zhejiang Normal University). https://doi.org/10.27464/d.cnki.gzsfu.2023.001940

[陈馨. (2023). *生物学POE教学策略对高中生批判性思维的影响研究* (硕士学位论文, 浙江师范大学). 硕士 https://link.cnki.net/doi/10.27464/d.cnki.gzsfu.2023.001940]

Chen, F. (2020). A study on the cooperative correction model in high school English writing and the cultivation of critical thinking ability (Master's thesis, Fujian Normal University). [https://doi.org/10.27019/d.cnki.gfjsu.2020.001517](https://doi.org/10.27019/d.cnki.gfjsu.2020.001517.)

[陈芳芳. (2020). *高中英语写作合作批改模式与批判性思维能力培养研究* (硕士学位论文, 福建师范大学). 硕士https://link.cnki.net/doi/10.27019/d.cnki.gfjsu.2020.001517]

Chen, J. (2017). An experimental study on cultivating critical writing skills of non-English major college students (Master's thesis, Northeast Normal University).

[陈建宇. (2017). *非英语专业大学生批判性写作能力培养实验研究* (硕士学位论文, 东北师范大学).]

Du, F. (2018). A study on the correlation between language proficiency and critical thinking ability of non-English major college students (Master's thesis, Hunan University).

[杜方超. (2018). *非英语专业大学生语言水平与思辨能力相关关系研究* (硕士学位论文, 湖南大学).]

Dong, T. (2018). *A study on the impact of problem-driven cooperative learning on high school students’ critical thinking* (Master’s thesis, Shanxi Normal University).

[董甜甜. (2018). *问题驱动下的合作学习对高中生批判性思维的影响研究* (硕士学位论文, 山西师范大学).]

Feng, Y. (2014). The construction of an interactive questioning model to promote college students' critical thinking and its empirical study (Master's thesis, Ningbo University).

[冯莹倩. (2014). *促进大学生批判性思维的提问交互模型构建及其实证研究* (硕士学位论文, 宁波大学).]

Fang, S. (2013). *A practical study on cultivating senior high school students’ critical thinking through the history of applied biological science* (Master’s thesis, Zhejiang Normal University). [https://doi.org/10.7666/d.Y2414305](https://doi.org/10.7666/d.Y2414305)

[方双双. (2013). *应用生物科学史培养高中生批判性思维的实践研究* (硕士学位论文, 浙江师范大学). https://doi.org/10.7666/d.Y2414305]

Gao, S. (2021). An empirical study on the impact of teacher questioning in high school English reading instruction on students' critical thinking (Master's thesis, Changchun Normal University). [https://doi.org/10.27709/d.cnki.gccsf.2021.000142](https://doi.org/10.27709/d.cnki.gccsf.2021.000142.)

[高姗姗. (2021). *高中英语阅读教学教师提问对学生批判性思维影响的实证研究* (硕士学位论文, 长春师范大学).硕士 [https://link.cnki.net/doi/10.27709/d.cnki.gccsf.2021.000142]](https://link.cnki.net/doi/10.27709/d.cnki.gccsf.2021.000142.])

He, W. (2021). A study on the impact of problem-driven and group learning-based integrated chemistry teaching on cultivating high school students' critical thinking (Master's thesis, Sichuan Normal University). <https://doi.org/10.27347/d.cnki.gssdu.2021.000367>

[何为. (2021). *基于问题驱动和小组学习的化学整合教学法对高中生批判性思维培养的研究* (硕士学位论文, 四川师范大学).硕士 [https://link.cnki.net/doi/10.27347/d.cnki.gssdu.2021.000367]](https://link.cnki.net/doi/10.27347/d.cnki.gssdu.2021.000367.])

Huang, X. (2021). A study on the application of English group reading in developing critical thinking among middle school students (Master's thesis, Sichuan Normal University). [https://doi.org/10.27347/d.cnki.gssdu.2021.000042](https://doi.org/10.27347/d.cnki.gssdu.2021.000042.)

[黄雪. (2021). *英语群文阅读在发展中学生批判性思维中的应用研究* (硕士学位论文, 四川师范大学). 硕士 [https://link.cnki.net/doi/10.27347/d.cnki.gssdu.2021.000042]](https://link.cnki.net/doi/10.27347/d.cnki.gssdu.2021.000042.])

Huang, C. (2022). *Teaching practice in senior high school biology for cultivating students’ critical thinking based on the PCRR model* (Master’s thesis, Guangxi Normal University).

[黄彩湄. (2022). *基于PCRR模型培养学生批判性思维的高中生物学教学实践研究* (硕士学位论文, 广西师范大学).]

Huang, Y. (2018). A study on the relationship between foreign language proficiency, cognitive control, and critical thinking ability among English major students (Master's thesis, Fujian Normal University).

[黄艳. (2018). *英语专业大学生外语水平、认知控制和思辨能力关系研究* (硕士学位论文, 福建师范大学).]

Jia, J. (2022). *Designing peer-assessment learning activities to promote the development of critical thinking* (Master’s thesis, Liaoning Normal University). https://doi.org/10.27212/d.cnki.glnsu.2022.000566

[贾佳. (2022). *促进批判性思维发展的同伴互评学习活动设计* (硕士学位论文, 辽宁师范大学).硕士 <https://link.cnki.net/doi/10.27212/d.cnki.glnsu.2022.000566>]

Jiang, J. Y. (2012). *A survey of Shanghai university students’ critical thinking and its relationship with personality* (Master’s thesis, Shanghai Normal University)

[蒋吉勇. (2012). *上海地区大学生批判性思维现状调查及其与人格的关系研究* (硕士学位论文, 上海师范大学).]

Jiang, X. (2018). *An empirical study on high school students’ critical thinking ability and English reading proficiency* (Master’s thesis, Zhejiang Normal University).

[姜昕苗. (2018). *高中生批判性思维能力及英语阅读水平的实证研究* (硕士学位论文, 浙江师范大学).]

Jin, Y., & Fang, F. (2014). An empirical study on the cultivation of critical thinking ability among English major students: Using feedback-based assessment in English writing as a medium. Journal of Hefei University (Social Sciences Edition), (06), 123–126.

[金玉 &方芳.(2014). 英语专业学生批判性思维能力培养的实证研究——以英语作文反馈式评价为媒介.*合肥学院学报(社会科学版)*(06), 123-126.]

Jou, M., Lin, Y. T., & Wu, D. W. (2016). Effect of a blended learning environment on student critical thinking and knowledge transformation. *Interactive Learning Environments*, *24*(6), 1131-1147.

Lei, L. (2021). A study on the impact of experiencing the physics experiment process on junior high school students' critical thinking skills (Master's thesis, Guangxi Normal University). [https://doi.org/10.27036/d.cnki.ggxsu.2021.001021](https://doi.org/10.27036/d.cnki.ggxsu.2021.001021.)

[雷凌湘. (2021). *亲历物理实验过程对初中生批判性思维技能的影响研究* (硕士学位论文, 广西师范大学). 硕士 [https://link.cnki.net/doi/10.27036/d.cnki.ggxsu.2021.001021]](https://link.cnki.net/doi/10.27036/d.cnki.ggxsu.2021.001021.])

Li, C., Yu, J., & Han, J. (2012). A survey and cultivation of critical thinking in English major students under the wave of innovation: A case study of Hebei Agricultural University. Journal of Hebei Agricultural University (Agricultural and Forestry Education Edition), (02), 56–59.

[李春燕, 于瑾 & 韩久全. (2012). 创新浪潮下英语专业学生批判性思维调查与培养——以河北农业大学为例. *河北农业大学学报(农林教育版)* (02), 56-59.]

Li, H. (2020). An empirical study on cultivating critical thinking ability in high school English reading teaching (Master's thesis, Anshan Normal University). [https://doi.org/10.27825/d.cnki.gassf.2020.000033](https://doi.org/10.27825/d.cnki.gassf.2020.000033.)

[李宏吉. (2020). *高中英语阅读教学批判性思维能力培养的实证研究* (硕士学位论文, 鞍山师范学院). 硕士 [https://link.cnki.net/doi/10.27825/d.cnki.gassf.2020.000033]](https://link.cnki.net/doi/10.27825/d.cnki.gassf.2020.000033.])

Liu, W. (2022). A practical study on cultivating high school students’ critical thinking ability based on a group cooperative learning model (Master’s thesis, Henan University). https://doi.org/10.27114/d.cnki.ghnau.2022.000642

[刘文娟. (2022). *基于小组合作学习模式培养高中生批判性思维能力的实践研究* (硕士学位论文, 河南大学).硕士 https://link.cnki.net/doi/10.27114/d.cnki.ghnau.2022.000642]

Lian, W. (2020). A study on the impact of reading-to-write on high school students' critical thinking ability (Master's thesis, Hebei Normal University). [https://doi.org/10.27110/d.cnki.ghsfu.2020.000437](https://doi.org/10.27110/d.cnki.ghsfu.2020.000437.)

[连维静. (2020). *以读促写对高中生批判性思维能力的影响研究* (硕士学位论文, 河北师范大学). 硕士 [https://link.cnki.net/doi/10.27110/d.cnki.ghsfu.2020.000437]](https://link.cnki.net/doi/10.27110/d.cnki.ghsfu.2020.000437.])

Liang, X. (2021). A study on the application of reading circles in college English major reading teaching (Master's thesis, Bohai University). [https://doi.org/10.27190/d.cnki.gjzsc.2021.000112](https://doi.org/10.27190/d.cnki.gjzsc.2021.000112.)

[梁雪飞. (2021). *阅读圈在大学英语专业阅读教学中的应用研究* (硕士学位论文, 渤海大学). 硕士 https://link.cnki.net/doi/10.27190/d.cnki.gjzsc.2021.000112 ]

Liao, Y. (2017). An empirical study on critical reading in high school English reading teaching (Master's thesis, Bohai University).

[廖一凡. (2017). *批判性阅读在高中英语阅读教学中的实证研究* (硕士学位论文, 渤海大学).]

Lin, J. (2014). Cultivating critical thinking in high school students through narrow reading (Master's thesis, Fujian Normal University). https://doi.org/10.27019/d.cnki.gfjsu.2014.000233

[林佳. (2014). *利用窄式阅读培养高中生批判性思维*(硕士学位论文, 福建师范大学).硕士 https://link.cnki.net/doi/10.27019/d.cnki.gfjsu.2014.000233 ]

Lin, Y. (2021). Design and application of "question chains" aimed at cultivating students' critical thinking in high school English reading teaching (Master's thesis, Fujian Normal University). [https://doi.org/10.27019/d.cnki.gfjsu.2021.001817](https://doi.org/10.27019/d.cnki.gfjsu.2021.001817.)

[林烨. (2021). *高中英语阅读教学中指向学生批判性思维培养的“问题链”设计与应用* (硕士学位论文, 福建师范大学). 硕士 https://link.cnki.net/doi/10.27019/d.cnki.gfjsu.2021.001817]

Ling, R. X. (2018). *Argumentation-based teaching practice in high school biology aimed at cultivating critical thinking* (Master’s thesis, Nanjing Normal University)

[凌荣秀. (2018). *基于批判性思维培养的高中生物论证式教学实践研究* (硕士学位论文, 南京师范大学).]

Liu, S. (2023). *Effects of teacher questioning in English reading instruction on high school students’ critical thinking* (Master’s thesis, Hunan Institute of Science and Technology). https://doi.org/10.27906/d.cnki.gnghy.2023.000060

[刘赛. (2023). *英语阅读教学中教师提问对高中生批判性思维的影响* (硕士学位论文, 湖南理工学院). 硕士 https://link.cnki.net/doi/10.27906/d.cnki.gnghy.2023.000060]

Lu, K. (2021). A study on the impact of question-based written feedback on cultivating high school students' critical thinking ability in English argumentative writing (Master's thesis, Qufu Normal University). [https://doi.org/10.27267/d.cnki.gqfsu.2021.000368](https://doi.org/10.27267/d.cnki.gqfsu.2021.000368.)

[卢克娟. (2021). *提问式书面反馈在英语议论文写作中对高中生批判性思维能力培养的影响研究* (硕士 学位论文, 曲阜师范大学). 硕士 https://link.cnki.net/doi/10.27267/d.cnki.gqfsu.2021.000368]

Luo, Q. X. (2002). *A study on the theory and assessment techniques of critical thinking* (Doctoral dissertation, Nanjing Normal University.)

[罗清旭. (2002). *批判性思维理论及其测评技术研究* (博士学位论文, 南京师范大学).]

Lü, Q. (2019). The impact of thematic content-based English reading instruction on high school students' critical thinking skills (Master's thesis, Northwest Normal University).

[吕倩倩. (2019). *主题式内容依托英语阅读教学对高中生思辨能力的影响* (硕士学位论文, 西北师范大学).]

Lv, X. (2020). *A study on senior high school English writing instruction based on the cultivation of critical thinking* (Master’s thesis, Nanjing Normal University).

[吕雪. (2020).*基于批判性思维培养的高中英语写作教学研究* (硕士学位论文, 南京师范大学).]

Miao, L. (2018). *A study on the cultivation of critical thinking ability in English writing among non-English major college students* (Master's thesis, Northwest University).

[苗丽华. (2018). *非英语专业大学生英语写作中思辨能力培养的研究* (硕士学位论文, 西北大学).]

Nie, Z. (2020). A practical study on cultivating critical thinking in high school information technology courses (Master's thesis, Bohai University). <https://doi.org/10.27190/d.cnki.gjzsc.2020.000405.>

[聂赵育. (2020). *高中信息技术课程中批判性思维培养的实践研究* (硕士学位论文, 渤海大学). 硕士 [https://link.cnki.net/doi/10.27190/d.cnki.gjzsc.2020.000405]](https://link.cnki.net/doi/10.27190/d.cnki.gjzsc.2020.000405.])

Ouyang, G. (2012). *A survey of the current development of critical thinking among Chinese college students and an exploration of influencing factors: A case study of H University* (Master’s thesis, Huazhong University of Science and Technology)

[欧阳光谱. (2012). *中国大学生批判性思维发展现状调查及影响因素探讨--以 H 大学为例* (硕士学位论文, 华中科技大学).]

Pan, Z. (2019). Action research on the types of teacher questioning in English reading classrooms to promote high school students' critical thinking (Master's thesis, Guangzhou University).

[潘智娴. (2019). *基于英语阅读课堂的教师提问类型促进高中生批判性思维的行动研究* (硕士学位论文, 广州大学).]

Peng, L. (2023). *Effects of a literature circles instructional model on high school students’ critical thinking* (Master’s thesis, Gannan Normal University). https://doi.org/10.27685/d.cnki.ggnsf.2023.000004

[彭柳. (2023). *阅读圈教学模式对高中生批判性思维的影响研究* (硕士学位论文, 赣南师范大学).硕士 https://link.cnki.net/doi/10.27685/d.cnki.ggnsf.2023.000004]

Qin, Y. (2014). An empirical study on effective methods to improve students' critical thinking ability in college English teaching (Master's thesis, Ocean University of China).

[秦玉清. (2014). *大学英语教学提高学生思辨能力的有效方法的实证研究* (硕士学位论文, 中国海洋大学).]

Qing, Z., Jing, G., & Yan, W. (2010). Promoting preservice teachers’ critical thinking skills by inquiry-based chemical experiment. *Procedia-Social and Behavioral Sciences*, *2*(2), 4597-4603.

Qing, Z., Xiang, W., & Linna, Y. A. O. (2007). A preliminary investigation into critical thinking of urban Xi'an high school students. *Frontiers of Education in China*, *2*(3), 447-468.

Su, F. (2020). A study on the impact of question types in high school English reading classrooms on students' critical thinking (Master's thesis, Northwest Normal University). [https://doi.org/10.27410/d.cnki.gxbfu.2020.001749](https://doi.org/10.27410/d.cnki.gxbfu.2020.001749.)

[苏粉艳. (2020). *高中英语阅读课堂提问类型对学生批判性思维影响的研究* (硕士学位论文, 西北师范大学). 硕士 https://link.cnki.net/doi/10.27410/d.cnki.gxbfu.2020.001749 ]

Su, Q. (2021). A study on follow-up questioning in high school English classrooms based on critical thinking cultivation (Master's thesis, Ningbo University). [https://doi.org/10.27256/d.cnki.gnbou.2021.001486](https://doi.org/10.27256/d.cnki.gnbou.2021.001486.)

[苏芩. (2021). *基于批判性思维培养的高中英语课堂追问研究* (硕士学位论文, 宁波大学). 硕士 [https://link.cnki.net/doi/10.27256/d.cnki.gnbou.2021.001486]](https://link.cnki.net/doi/10.27256/d.cnki.gnbou.2021.001486.])

Sun, S. (2021). A study on the correlation between the cognitive levels of teacher questioning and high school students' critical thinking ability (Master's thesis, Hebei Normal University). <https://doi.org/10.27110/d.cnki.ghsfu.2021.001165>

[孙硕. (2021). *教师提问认知层级与高中生批判性思维能力的相关性研究* (硕士学位论文, 河北师范大学). 硕士 https://link.cnki.net/doi/10.27110/d.cnki.ghsfu.2021.001165]

Tang, Y. (2020). A study on the correlation between high school students' critical thinking ability and English reading ability (Master's thesis, Nanning Normal University). [https://doi.org/10.27037/d.cnki.ggxsc.2020.000122](https://doi.org/10.27037/d.cnki.ggxsc.2020.000122.)

[唐杨. (2020). *高中生思辨能力与英语阅读能力相关性研究* (硕士学位论文, 南宁师范大学). 硕士 https://link.cnki.net/doi/10.27037/d.cnki.ggxsc.2020.000122 ]

Tian, H. (2009). The impact of task-driven cooperative learning in chemistry experimental teaching on high school students' critical thinking (Master's thesis, Shaanxi Normal University).

[田红. (2009). *基于任务驱动的合作学习的化学实验教学对高中生批判性思维的影响* (硕士学位论文, 陕西师范大学).]

Tong, J. (2021). *A study on the practice of problem-driven cooperative learning to promote high school students' critical thinking* (Master's thesis, Northwest Normal University). [https://doi.org/10.27410/d.cnki.gxbfu.2021.001036](https://doi.org/10.27410/d.cnki.gxbfu.2021.001036.)

[童佳欣. (2021). *促进高中生批判性思维的问题驱动下的合作学习实践研究* (硕士学位论文, 西北师范大学). 硕士 https://link.cnki.net/doi/10.27410/d.cnki.gxbfu.2021.001036 ]

Wang, H. (2016). *The effects of a concept-mapping instructional strategy on high school students’ critical thinking* (Master’s thesis, Shanxi Normal University)

[王慧婷. (2016). *概念图教学策略对高中生批判性思维的影响* (硕士学位论文, 山西师范大学).]

Wang, W. (2021). A comparative study of critical thinking skills between English and Business English majors. Journal of Jingdezhen University, (01), 51–55.

[王伟. (2021). 英语与商务英语专业学生思辨技能的比较研究. *景德镇学院学报*(01), 51-55.]

Wang, Y. (2008). The current status of critical thinking in chemistry education majors and the impact of inquiry-based experiments (Master's thesis, Shaanxi Normal University).

[王燕. (2008). *化学师范生批判性思维现状及探究性实验对其的影响* (硕士学位论文, 陕西师范大学).]

Xiao, Y., & Tan, J. (2020). Design and practice of online peer assessment learning activities to enhance college students' critical thinking ability: A case study of the climate monitoring course. Journal of Guangxi Vocational and Technical College, (03), 63–69.

[肖艳丹 & 谭姣连. (2020). 提升大学生批判性思维能力的在线同伴评估学习活动的设计与实践——以气候监测课程为例. *广西职业技术学院学报*(03), 63-69.]

Xu, J. (2020). *Designing evaluation activities in blended learning to promote the development of critical thinking* [Master’s thesis, Northwest Normal University] . https://doi.org/10.27410/d.cnki.gxbfu.2020.001326

[徐娟莉. (2020). *促进批判性思维发展的混合学习评价活动设计研究* (硕士学位论文, 西北师范大学). 硕士 https://link.cnki.net/doi/10.27410/d.cnki.gxbfu.2020.001326 doi:10.27410/d.cnki.gxbfu.2020.001326]

Xu, P. (2010). Preliminary study on primitive chemical questions (Master's thesis, Shaanxi Normal University).

[徐盼盼. (2010). *原始化学问题的初步研究* (硕士学位论文, 陕西师范大学).]

Xu, J. (2023). *An online learning intervention to promote critical thinking development* (Master’s thesis, Northwest Normal University). https://doi.org/10.27410/d.cnki.gxbfu.2023.001409

[徐骥轩. (2023). *促进批判性思维发展的在线学习干预研究* (硕士学位论文, 西北师范大学).硕士 https://link.cnki.net/doi/10.27410/d.cnki.gxbfu.2023.001409]

Yao, L. N. (2006). *Critical thinking and its implications for chemistry teaching* (Master’s thesis, Shaanxi Normal University)

[姚林娜. (2006). *批判性思维及其对化学教学的意义* (硕士学位论文, 陕西师范大学).]

Yang, W. (2018). A study on the impact of experiential learning in high school biology on students' critical thinking (Master's thesis, Guizhou Normal University).

[杨雯芮. (2018). *高中生物体验式学习对学生批判性思维的影响研究* (硕士学位论文, 贵州师范大学).]

Yang, L. (2007). *Cultivating critical thinking in senior high school chemistry teaching* (Master’s thesis, Shaanxi Normal University).

[杨玲. (2007).*高中化学教学中批判性思维培养的研究* (硕士学位论文,陕西师范大学).]

Yang, Y. (2022). A study on the teaching model for cultivating preservice teachers' critical thinking from the TPACK perspective (Master's thesis, Hebei Normal University). [https://link.cnki.net/doi/10.27110/d.cnki.ghsfu.2022.000765](https://link.cnki.net/doi/10.27110/d.cnki.ghsfu.2022.000765)

[杨艺朋. (2022). *TPACK视角下师范生审辨思维培养教学模式研究* (硕士学位论文, 河北师范大学). 硕士 https://link.cnki.net/doi/10.27110/d.cnki.ghsfu.2022.000765 ]

Yang, Y. T. C., & Chou, H. A. (2008). Beyond critical thinking skills: Investigating the relationship between critical thinking skills and dispositions through different online instructional strategies. *British Journal of Educational Technology*, *39*(4), 666-684.

Yang, Y. T. C., Chuang, Y. C., Li, L. Y., & Tseng, S. S. (2013). A blended learning environment for individualized English listening and speaking integrating critical thinking. *Computers & Education*, *63*, 285-305.

Ye, J. (2021). *An experimental study on the impact of deep reading instruction on junior high school students' critical thinking* (Master's thesis, Guangzhou University). [https://link.cnki.net/doi/10.27040/d.cnki.ggzdu.2021.000131](https://link.cnki.net/doi/10.27040/d.cnki.ggzdu.2021.000131)

[叶健萍. (2021). *英语深度阅读教学对初中生批判性思维影响的实验研究* (硕士学位论文, 广州大学). 硕士 https://link.cnki.net/doi/10.27040/d.cnki.ggzdu.2021.000131]

Yuan, H. (2012). *Construction of a model for questioning suggestibility and its influencing factors* (Doctoral dissertation, Central South University).

[袁红梅. (2012). *质疑暗示感受性及其影响因素模型建构*  (博士学位论文, 中南大学).]

Yuan, H., Kunaviktikul, W., Klunklin, A., & Williams, B. A. (2008). Improvement of nursing students' critical thinking skills through problem‐based learning in the People's Republic of China: A quasi‐experimental study. *Nursing & health sciences*, *10*(1), 70-76.

Zhang, R. (2017). *A study on the application of critical reading strategies in junior high school English reading teaching* (Master's thesis, Liaoning Normal University).

[张冉. (2017). *批判性阅读策略应用于初中英语阅读教学的研究* (硕士学位论文, 辽宁师范大学).]

Zhang, Y. (2018). A study on the development of critical thinking ability in high school English reading and writing teaching (Master's thesis, Central China Normal University). [https://doi.org/10.27159/d.cnki.ghzsu.2018.000085](https://doi.org/10.27159/d.cnki.ghzsu.2018.000085.)

[张源瑞. (2018). *“高中英语读写教学中思辨能力发展的研究* (硕士学位论文, 华中师范大学). 硕士 https://link.cnki.net/doi/10.27159/d.cnki.ghzsu.2018.000085]

Zhang, Y. (2022). A study on the application of critical reading in high school English reading teaching (Master's thesis, Xinyang Normal University). [https://doi.org/10.27435/d.cnki.gxsfc.2022.000492](https://doi.org/10.27435/d.cnki.gxsfc.2022.000492。)

[张瑜颖. (2022). *批判性阅读在高中英语阅读教学中的应用研究* (硕士学位论文, 信阳师范学院). 硕士 [https://link.cnki.net/doi/10.27435/d.cnki.gxsfc.2022.000492]](https://link.cnki.net/doi/10.27435/d.cnki.gxsfc.2022.000492.])

Zhao, J. (2012). The impact of cross-disciplinary online chatting on the thinking of English major students (Master's thesis, Shandong Normal University).

[赵吉龙. (2012). *跨专业网聊对英语专业学生思维的影响* (硕士学位论文, 山东师范大学).]

Zhong, L. (2019). A practical study on cultivating students' critical thinking in high school biology teaching (Master's thesis, Fujian Normal University). [https://doi.org/10.27019/d.cnki.gfjsu.2019.001755](https://doi.org/10.27019/d.cnki.gfjsu.2019.001755.)

[钟磊发. (2019). *高中生物学教学中培养学生批判性思维的实践研究* (硕士学位论文, 福建师范大学). 硕士 https://link.cnki.net/doi/10.27019/d.cnki.gfjsu.2019.001755]

Zhou, Q., Yan, C., Zhao, S., Liu, L., & Xing, L. (2012, December). A preliminary investigation into critical thinking of in-service and pre-service middle school chemistry teachers in Shaanxi province of China. *In Asia-Pacific Forum on Science Learning and Teaching* (Vol. 13, No. 2, pp. 1-13). The Education University of Hong Kong, Department of Science and Environmental Studies.

Zhou, Z., & Han, C. (2022). A survey on critical thinking among master's degree students in English education. Journal of Jimei University (Education Science Edition), (04), 36–42.

[周志成 & 韩存新. (2022). 英语教育硕士研究生批判性思维调查研究. *集美大学学报(教育科学版)*(04), 36-42.]

Zhou, D. (2023). *An empirical study on the impact of learning-sheet teaching in senior high school biology on students’ critical thinking* (Master’s thesis, Zhejiang Normal University).

[周丹阳.(2023). *高中生物学历案教学对学生批判性思维影响的实证研究* (硕士学位论文, 浙江师范大学).]

Zhou, J. (2022). *Cultivating high school students’ critical thinking driven by problem chains* (Master’s thesis, Mudanjiang Normal University). https://doi.org/10.27757/d.cnki.gmdjs.2022.000324

[周剑. (2022). *问题链驱动高中生批判性思维培养的研究* (硕士学位论文, 牡丹江师范学院). 硕士 https://link.cnki.net/doi/10.27757/d.cnki.gmdjs.2022.000324]

Zhu, X. L., Zheng, Q., Xu, F. F., Yang, Y. Q., Li, Y. M., & Wu, Y. Q. (2020). Analysis of the effect of a case-design teaching method on improving undergraduates’ critical thinking ability . *Journal of Wenzhou Medical University, 50*(8), 686–689.

[朱晓玲, 郑琼, 许芳芳, 杨晔琴, 李一美 & 吴永琴. (2020). 案例设计教学法对提高本科生批判性思维能力的效果分析. *温州医科大学学报, 50*(08), 686-689.]

Zhu, M. (2021). A study on the application of critical thinking skills in high school English reading teaching (Master's thesis, Hunan Normal University). [https://doi.org/10.27137/d.cnki.ghusu.2021.002768](https://doi.org/10.27137/d.cnki.ghusu.2021.002768.)

[朱芒芒. (2021). *批判性思维技能在高中英语阅读教学中的应用研究* (硕士学位论文, 湖南师范大学). 硕士 https://link.cnki.net/doi/10.27137/d.cnki.ghusu.2021.002768 ]

Zhuo, X. (2019). Using STEM courses to cultivate students' dialectical thinking (Master's thesis, Guangxi Normal University). [https://doi.org/10.27036/d.cnki.ggxsu.2019.000050](https://doi.org/10.27036/d.cnki.ggxsu.2019.000050.)

[卓雪妹. (2019).*用STEM课程培养学生审辩式思维* (硕士学位论文, 广西师范大学). 硕士 https://link.cnki.net/doi/10.27036/d.cnki.ggxsu.2019.000050]

Zong, J. (2018). An experimental study on the application of argumentation mind maps in college English argumentative writing teaching (Master's thesis, Northeast Normal University).

[宗佳菊. (2018). *思维论证导图在大学英语议论文写作教学中应用的实验研究* (硕士学位论文, 东北师范大学).]
